# From Structure to Function: The Impact of EGFR and IGF-IR in 3D Breast Cancer Spheroids

**DOI:** 10.3390/cancers17162606

**Published:** 2025-08-08

**Authors:** Chrisavgi Gourdoupi, Spyros Kremmydas, Sylvia Mangani, Paraskevi Ioannou, Nikolaos A. Afratis, Zoi Piperigkou, Nikos K. Karamanos

**Affiliations:** 1 Biochemistry, Biochemical Analysis & Matrix Pathobiology Research Group, Laboratory of Biochemistry, Department of Chemistry, University of Patras, 26504 Patras, Greece; up1061190@ac.upatras.gr (C.G.); up1073639@ac.upatras.gr (S.K.); sylvia.mangani@upatras.gr (S.M.); 2 Laboratory of Biochemistry, Biotechnology and Molecular Analysis, Department of Agricultural Development, Agri-Food & Management of Natural Resources, National and Kapodistrian University of Athens, Evripos Campus, 34400 Psachna, Evia, Greece; ioannouevi@agro.uoa.gr (P.I.); nafratis@agro.uoa.gr (N.A.A.)

**Keywords:** breast cancer, EGFR, IGF-IR, 2D and 3D cell models, spheroids, extracellular matrix, matrix metalloproteinases

## Abstract

**Simple Summary:**

This study highlights the importance of evaluating cancer cells’ functional properties and gene expression in 3D breast cancer cell models over traditional 2D culture systems to mimic the tumor environment better so as to determine more efficient therapeutic approaches. By examining triple-negative and luminal A breast cancer subtypes, this research evaluates how the inhibition of the EGFR and IGF-IR pathways—separately and in combination—affects cell behavior and extracellular matrix expression. Our results revealed potential crosstalk between these pathways, influencing key functions such as cell proliferation, migration, and dissemination. Significant results in 3D models were observed, especially in the expression of matrix metalloproteinases. In summary, our findings emphasize the crucial role of EGFR and IGF-IR interactions in breast cancer progression and demonstrate the importance of using physiologically relevant 3D cell platforms in cancer research.

**Abstract:**

**Background**: Breast cancer, one of the most researched cancers in oncology, remains the primary cause of cancer-related mortality in women. Its biological complexity, which includes phenotypic, genetic, and microenvironmental aspects, makes modeling and treatment quite difficult. The need for more physiologically realistic models is highlighted by the comparison of two-dimensional (2D) cell cultures with 3D breast-cancer-derived spheroids, which discloses how important pathways such as epidermal growth factor receptor (EGFR) and insulin-like growth factor I receptor (IGF-IR) influence cell behavior and extracellular matrix (ECM) macromolecular expression. **Methods**: The purpose of this study was to utilize novel 3D cell platforms to assess the effect of inhibiting the EGFR and IGF-IR pathways, alone or in combination, on the functional properties and the expression levels of certain matrix metalloproteinases (MMPs) which are implicated in breast cancer progression (i.e., triple-negative and luminal A breast cancer subtypes) and related with the EGFR and IGF-ΙR molecular network, as also demonstrated through STRING analysis. **Results**: Our results demonstrated potential crosstalk between EGFR and IGF-IR signaling, which influences cell proliferation and spheroid growth, dissemination, and migration. Significant phenotypic changes proposed between 2D and 3D cell cultures, and alterations in the expression of MMPs, were also recorded. **Conclusions**: Both breast cancer cell lines retained acknowledged characteristics across the tested models while also exhibiting new, condition-dependent properties. Overall, our findings enhance our understanding on the interplay between the EGFR and IGF-IR pathways and underscore the value of 3D models in revealing key biological processes underlying distinct breast cancer phenotypes.

## 1. Introduction

Breast cancer is a complex and heterogenous disease with a multifactorial evolutionary profile. It arises from epithelial cells found within the mammary ducts or lobules, and its progression involves a multistep process characterized by genetic, epigenetic, and microenvironmental alterations that contribute to uncontrolled cell proliferation, tissue invasion, and distant metastasis [1]. Recent advancements in molecular profiling have enabled the classification of breast cancer into intrinsic subtypes according to gene expression patterns, providing clinical significance for prognosis and treatment decisions. These subtypes comprise luminal A, luminal B, HER2-enriched, and triple-negative breast cancer (TNBC). Luminal A tumors are generally estrogen receptor (ERα)-positive and HER2-negative, displaying low rates of proliferation and favorable clinical outcomes. Conversely, TNBC is marked by the absence of ERα, progesterone receptor (PR), and HER2, correlating with a high histological grade, greater metastatic potential, and limited treatment alternatives. The biological aggressiveness and poor outlook of TNBC underscore the need to investigate new therapeutic targets and enhance our understanding of the cellular and molecular mechanisms involved in tumor progression [2,3]. 

A crucial component of breast cancer development involves not only the malignant cells but also their surrounding environment, referred to as the tumor microenvironment (TME). The TME consists of various cell populations, including fibroblasts, immune cells, endothelial cells, and stromal ECM components. The ECM serves as a dynamic environment comprising structural proteins such as collagen, fibronectin, and laminin, as well as different proteoglycans, offering both biochemical and mechanical signals to neighboring and invading tumor cells [4]. The remodeling of the ECM is a prominent feature of cancer development and is thoroughly controlled by enzymes like MMPs, which degrade ECM components to facilitate invasion and metastasis. Particularly, MMP-14 and MMP-9 have been linked to the migration, angiogenesis, and metastasis of breast cancer cells [5]. The interaction between breast cancer cells and the ECM is a dynamic process; it involves reciprocal signaling that affects tumor cell behavior, adaptability, and resistance to therapies. These interactions are frequently disrupted in aggressive subtypes like TNBC, where altered MMP expression and ECM disorganization are noted, leading to a favorable environment for tumor spread [6].

Receptor tyrosine kinases (RTKs) are essential mediators of cellular signaling and play vital roles in controlling key oncogenic processes such as proliferation, survival, movement, and differentiation [7]. Among these RTKs, the epidermal growth factor receptor (EGFR) and insulin-like growth factor I receptor (IGF-IR) are often associated with breast cancer. EGFR is frequently overexpressed in TNBC, where its activation promotes pro-tumorigenic pathways like RAS/RAF/MEK/ERK and PI3K/AKT/mTOR [8]. Likewise, IGF-IR signaling, activated by its ligands IGF-I and IGF-II, influences downstream pathways that encourage cell growth and prevent apoptosis. Abnormal activation of these receptors is linked to a poor prognosis and resistance to conventional therapies [9,10,11].

Tyrosine kinase inhibitors (TKIs) represent an essential class of small molecules frequently utilized in targeted cancer therapies due to their capacity to obstruct abnormal kinase signaling pathways that facilitate tumor growth and survival. TKIs can be categorized according to their chemical structure, binding mechanism, clinical use, and generation [12]. From a structural perspective, they encompass derivatives like aminopyrimidines, quinolines, and quinazolines. In terms of mechanism, TKIs are classified as either reversible, which bind noncovalently to the ATP-binding site, or irreversible, which create covalent bonds with specific amino acid residues [13,14]. Among the numerous TKIs developed, AG1478 and AG1024 are two well-studied aromatic compounds employed in preclinical evaluations. AG1478 is a strong, reversible inhibitor of EGFR, with its effectiveness linked to its quinazoline-based structure and hydrophobic interaction [15]. AG1024, a reversible inhibitor of IGF-IR, inhibits crucial downstream pathways such as MAPK and Akt, leading to decreased cell proliferation. Both inhibitors serve as important tools for examining receptor-specific signaling in cancer models and are frequently used to evaluate therapeutic approaches, especially in tumors that exhibit resistance to conventional treatments [16].

While traditional 2D cell culture systems have been important in biomedical research because of their simplicity and reproducibility, they do not effectively mimic the complex structure and environment of in vivo tumors [17]. They cannot describe key cell–cell and cell–matrix interactions that affect cellular behaviors like growth, differentiation, gene expression, and cell death [18]. In 2D cultures, cells often lose their natural morphology and polarization, which results in changed signaling and biological responses. Moreover, the equal exposure to nutrients, oxygen, and drugs in monolayer cultures does not reflect the varied conditions found in solid tumors [19]. In contrast, 3D culture models, like spheroids and organoids, resemble better the TME. These structures include different zones—an outer layer of growing cells, a middle region of quiescent cells, and a hypoxic core—mirroring the physiological gradients in tumors. Therefore, 3D models provide a more relevant way to study tumor-specific processes, including migration, the epithelial-to-mesenchymal transition (EMT), and metastasis [20,21]. Overall, 3D spheroid systems offer clear benefits for understanding tumor biology and creating more effective, targeted cancer treatments.

This research is therefore focused on two primary aims: (i) to illustrate the enhanced biological relevance of 3D cell culture models in mimicking the features of the TME compared to that of the conventional 2D systems and (ii) to evaluate the roles of EGFR and IGF-IR in the behavior of breast cancer cells and the expression of major MMPs using the respective downstream inhibitors, AG1478 and AG1024. By examining the impact of these RTK inhibitors on both MDA-MB-231 (TNBC) and MCF-7 (luminal A) cells in both 2D and 3D models, this study sought to gain deeper insight into the mechanisms driving cancer progression and to determine potential molecular targets for therapeutic strategies.

## 2. Materials and Methods

### 2.1. Cell Cultures and Reagents

Luminal A (ERα-positive, low metastatic potential) MCF-7 and TNBC (ERα-negative/ERβ-positive, high metastatic potential) MDA-MB-231 cell lines were purchased from the American Type Culture Collection (ATCC, Manassas, VA, USA). Both breast cancer cell lines were cultured at 37 °C in an atmosphere of 5% CO_2_ and 95% air in Dulbecco’s Modified Eagle Medium (DMEM, LM-D1110/500, Biosera, France) complete cell culture medium. The culture medium was supplemented with 100 IU/mL of penicillin, 100 μg/mL of streptomycin, 10 μg/mL of gentamycin sulfate, 2.5 μg/mL of amphotericin B, 1 mM of sodium pyruvate, and 2 mM of L-glutamine (XC-T1715/100, Biosera, France), and 10% fetal bovine serum (FBS, FB-1000/500, Biosera, France). When the cells reached approximately 80–85% confluency, they were sub-cultivated using 1x trypsin–EDTA in PBS (LM-T1706/500, Biosera, France). Every experiment used three distinct biological replicates and was carried out in starvation conditions. Cytarabine, a cytostatic drug, was acquired from Sigma-Aldrich in Saint Louis, Missouri, USA. Dimethyl sulfoxide (DMSO) was used to prepare the stock solutions of the inhibitors Tyrphostin AG1478 (658552, Sigma Aldrich, Merck, Darmstadt, Germany) and Tyrphostin AG1024 (121767, Sigma Chemical Co., St Louis, MO, USA). Treatments with the inhibitors were carried out in serum-free conditions to avoid net effects and to exclude estrogenic effects on our experimental setups. Based on previous data from our group [22,23], the working concentrations of AG1478 and AG1024 were 2 μM and 1 μΜ in DMEM with 0% FBS, respectively.

### 2.2. RNA Isolation, cDNA Synthesis, and Real-Time qPCR Analysis in 3D Conditions

Both breast cancer cell lines were plated into ultra-low-adhesion 96-well plates (83.3925.400, SARSTEDT, Nümbrecht, Germany) at a density of 15,000 cells per well. Th cells were left in complete medium for 3 days to form spheroids and then starved with serum-free medium (0% FBS) for 16–20 h. Then, the inhibitors [AG1478 (2 μΜ), AG1024 (1 μΜ), and a mixture of both (AG1478 + AG1024] were added to the serum-free medium, and the spheroids were incubated for 24 h. On the next day, the spheroids were harvested and stored at −80 °C. Using the NucleoSpinRNA II Kit (Macherey-Nagel, Duren, Germany), total RNA was extracted from the cells. The absorbance of the RNA extracted at 260 nm was used to quantify it. By analyzing 260/280 nm and 260/230 nm ratios of each RNA extract, RNA purity was guaranteed. Using KAPA Taq Ready Mix DNA Polymerase (KAPA BIOSYSTEMS, Wilmington, MA, USA) and the PrimeScript TM1st strand cDNA ideal real-time synthesis kit (Takara Bio Inc., Goteborg, Sweden), the total RNA was reverse-transcribed. Following the manufacturer’s instructions, a real-time PCR analysis was performed. The Rotor Gene Q was used for amplification (Qiagen, Germantown, MD, USA). Every reaction was carried out in triplicate, and for assay validation, a standard curve was always provided for every pair of primers. A fluorescence threshold above the background was set to determine the threshold cycle (Ct) number, which corresponded to the point during the early logarithmic phase of amplification where product accumulation became detectable for measurement. Using the ΔΔCt method, the relative expressions of several gene transcripts were determined. The Ct values of each one of the genes of interest were normalized to the Ct value of the housekeeping gene. To ensure accurate normalization of the real-time qPCR data, we followed the MIQE (Minimum Information for Publication of Quantitative Real-Time PCR Experiments) guidelines, and we initially assessed both the *GAPDH* and *ACTB* housekeeping genes, which were confirmed to exhibit stable expression across all experimental conditions. For clarity and to avoid redundancy in the data presentation, the qPCR results were normalized using *ACTB* alone, as its expression remained consistently stable and representative of the normalization strategy employed. Fold changes were calculated as 2^−ΔΔCt^ (arbitrary units). Detailed information about the target genes, as well as the primers utilized, is listed in Table 1.

### 2.3. The Proliferation Assay in 2D Conditions

MCF-7 and MDA-MB-231 cells were seeded into 96-well plates at a density of 7500 and 5000 cells per well, respectively. Following a 24 h incubation period in complete medium, as previously mentioned, the media was switched to serum-free, and the cells were starved for the whole night. The cells were cultured for 24 h after the addition of the inhibitors AG1478 (2 μM) and AG1024 (1 μΜ) and a combination of both (AG1478 + AG1024) to the serum-free medium (0% FBS). To evaluate the effect of the TKIs on cell proliferation, the WST-1 assay was used. Briefly, 10 μL of WST-1 reagent was added to each well. Following 1 h of incubation, the plates were transferred onto a bench rocker for 5 min in the dark to prevent light degradation. Finally, a Tecan photometer (with a reference wavelength at 650 nm) was used to quantify each well’s optical density/absorbance at 450 nm. For all measurements, the absorbance of a blank sample was recorded and subtracted to correct for the background signal. Subsequently, the values of the experimental samples were normalized to the control conditions.

### 2.4. The Spheroid Growth Assay

At a density of 10,000 cells per well, MCF-7 and MDA-MB-231 cells were seeded into 96 round-bottom ultra-low-adhesion well plates (SARSTEDT, Nümbrecht, Germany). Until spheroids formed, the cells were cultivated for three days in an enriched medium without any media changes. The cells were then starved overnight after the medium was switched to serum-free. After fasting, the spheroids were cultured for 24 h in a serum-free medium containing the inhibitors AG1478 (2 μM) and AG1024 (1 μΜ) and a combination of both (AG1478 + AG1024). The control for this assay was untreated spheroids in serum-free (0% FBS) conditions. Photos were taken at every step to monitor the development of the spheroids, as well as their growth in the presence of the inhibitors.

### 2.5. The Dissemination Assay

To investigate spheroid dissemination, the spheroids were transferred into regular 96-well plates (1 spheroid per well), and photos were taken at regular intervals at 24, 48, 72, and 96 h for the MCF-7- and at 24, 48, and 72 h for the MDA-MB-231-derived 3D spheroids. Note that at 96 h, all of the disseminated cells that came from the MDA-MB-231-derived spheroids had spread all over the culture well. The spheroids were transferred with care using a pipette fitted with cut tips to prevent mechanical damage. The photos were further analyzed using ImageJ (version 1.50b Launcher Symmetry Software, LOCI, University of Wisconsin, WI, USA) to quantify the area of dissemination and the tumor core utilizing the ‘Polygon Selections’ tool, followed by the ‘Analyze’ and ‘Measure’ functions. The dissemination core is defined as the area covered by cells as they migrate away from the spheroid mass, whereas the tumor core refers to the highly compact region of cells within the spheroid. The tumor core and dissemination area were measured in inches^2^, and the scale was 300 pixels/inch. All of the data were subsequently normalized to the corresponding values at 0 h for each experimental condition. As a result of this normalization, the units are effectively canceled, and the control is assigned a relative value of 1.

### 2.6. The Wound Healing Assay in 3D Conditions

To assess spheroid migratory capacity, 5 spheroids from each treatment group were transferred into individual wells in a 48-well plate, where they were allowed to spread until they covered the well surface. In both assays, the spheroid-derived cells were replated into the new wells in DMEM supplemented with 5% FBS. After 6 days for MCF-7 and 4 days for MDA-MB-231, the wound healing assay was conducted. Specifically, following 4 h of starvation with serum-free medium, a cross-pattern was scratched into the cell monolayer using a sterile 100 μL pipette tip. To remove any unattached cells, each well was gently rinsed twice with 1x PBS. The cytostatic drug cytarabine (10 μM) was then added to the serum-free media to prevent cell growth and guarantee that cell migration alone was responsible for the reported outcomes. The inhibitors AG1478 (2 μΜ) and AG1024 (1 μΜ) and their combination (AG1478 + AG1024) were administered to the serum-free medium following a 30-min incubation period. At 0, 24, and 48 h, the wound closure was documented using a digital camera attached to a phase-contrast microscope. The impact of the inhibitors and their combination on cancer cell migration was assessed by quantifying the wound surface area using an image analysis (ImageJ version 1.50b Launcher Symmetry Software, LOCI, University of Wisconsin, WI, USA). This was performed using the ‘Polygon Selections’ tool, followed by the ‘Analyze’ → ‘Measure’ functions, and the same parameters described above in Section 2.5.

### 2.7. The STRING Database

The STRING database is part of the ELIXIR Core Data Resources and is a well-known database that predicts protein–protein interactions. These interactions include both direct (physical) and indirect (functional) associations, derived from computational predictions, knowledge transfer between organisms, and interactions compiled from other primary databases. The five main sources of data are (a) Genomic Context Predictions, (b) High-throughput Laboratory Experiments, (c) (Conserved) Co-expression, (d) Automated Text Mining, and Previous Knowledge in Databases. The interactions presented in the STRING network may represent either known associations supported by published experimental evidence or predicted interactions inferred through computational methods, such as text mining, a co-expression analysis, or protein homology [24]. The process followed in this study was as follows: the selection of the “Multiple proteins” tool and input of the protein names and the studied organism, followed by the Legend section.

### 2.8. The Human Protein Atlas

The Human Protein Atlas (https://www.proteinatlas.org, accessed on 20 May 2025) proteome analysis resource is based in Sweden and was launched in 2003, with the goal of mapping all human proteins found in cells, tissues, and organs through the integration of various omics technologies, which include antibody-based imaging, mass-spectrometry-based proteomics, transcriptomics, and systems biology. All information within this knowledge resource is publicly accessible, enabling researchers in both academic and industrial settings to freely explore the data related to the human proteome [25]. The resource that was used was “Cell line”.

### 2.9. Statistical Analysis

The data are presented as the mean ± standard deviation (SD) from experiments performed in triplicate. The statistical significance was assessed using an analysis of variance (ANOVA) test. For the post hoc analysis, Tukey’s test was employed to adjust for multiple pairwise comparisons within each factor, ensuring control over the family-wise error rate within each assay. This test was used to identify statistically significant differences among the four groups (control, AG1478, AG1024, and combination treatment). Statistical significance was defined as *p* ≤ 0.05, *p* ≤ 0.01, and *p* ≤ 0.001, indicated by (*, **, ***) for comparisons between the treatment and control groups and by a hash sign (#, ##, ###) for comparisons between treatment groups. All statistical analyses and graphical representations were performed using GraphPad Prism version 8.0.1 (GraphPad Software, San Diego, CA, USA).

## 3. Results

### 3.1. The Effects of TKIs on MDA-MB-231 and MCF-7 Proliferation in 2D Cell Culture Conditions

Proliferation assays assist researchers in evaluating cancer growth [26]. MDA-MB-231 and MCF-7 are two breast cancer cell lines exhibiting distinct phenotypic characteristics. MDA-MB-231 has mesenchymal features and is related to more aggressive tumors. MCF-7 is a low-invasive, hormone-dependent cell line, distinguished by its epithelial-like phenotype [27]. Therefore, it is reasonable to examine the effects of EGFR and IGF-IR on cancer cell proliferation, considering the important role of these RTKs in breast cancer. To evaluate the effect of EGFR and IGFR-IR on the proliferation of the MCF-7 and MDA-MB-231 cell lines, the cells were treated with the respective inhibitors AG1478 and AG1024 and a mixture of both (AG1478 + AG1024) at final concentrations of 2 μΜ and 1 μM, respectively [28,29].

**Figure 1:**
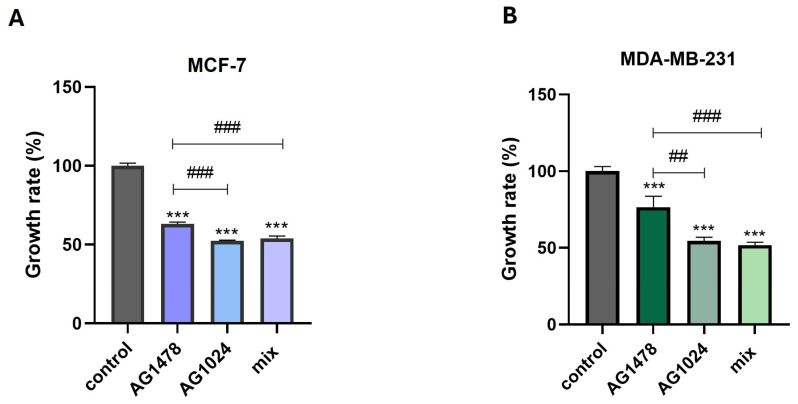
The effects of EGFR and IGF-IR on breast cancer cell growth. (**A**) A quantification graph of MCF-7 proliferation in the presence of AG1478, AG1024, and their mix following 24 h of treatment. (**B**) A quantification graph of MDA-MB-231 in the presence of AG1478, AG1024, and their mix following 24 h of treatment. Each bar represents the mean ± SD values. An asterisk (*) indicates statistically significant differences (*p* < 0.05), two asterisks indicate statistically significant differences *p* < 0.01, and three asterisks indicate statistically significant differences (*p* < 0.001) compared to the control samples. A hash (#) indicates statistically significant differences (*p* < 0.05), two hashes indicate statistically significant differences (*p* < 0.01), and three hashes indicate statistically significant differences (*p* < 0.001) among the different samples.

In the current study, the inhibition of EGFR and IGF-IR and their dual inhibition in MCF-7 led to a significant decrease in cell proliferation (Figure 1A). Specifically, AG1478 decreases the cell growth to *ca* 35%, AG1024 decreases it to *ca* 50%, and their combination decreases it to *ca* 50%. Notably, the proliferation rate of the MDA-MB-231 cells following IGF-IR inhibition was significantly decreased (*ca* 55%) as compared to that in the control cells (untreated cells in serum-free conditions), indicating the additional importance of the IGF-IR signaling pathway in sustaining cell growth in these cells (Figure 1B). The importance of these receptor-dependent pathways is more obvious in the sample with the combination of both inhibitors, with the highest effect reaching a *ca* 55% reduction in cell growth. These findings support the significance of EGFR and highlight the contribution of IGF-IR signaling in both cell lines.

### 3.2. The Effect of TKIs on the Growth Size of Breast-Cancer-Cell-Derived Spheroids

**Figure 2:**
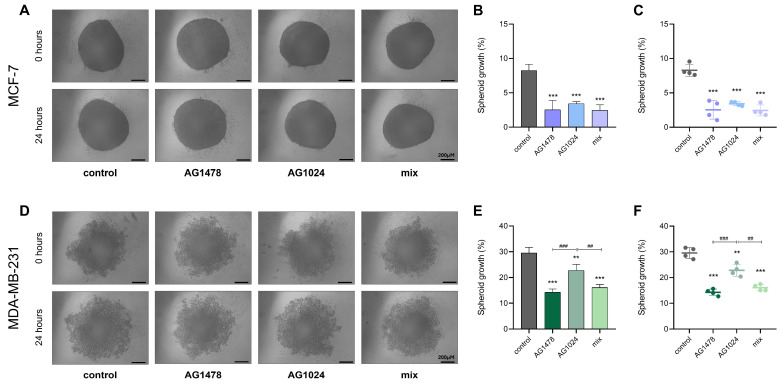
The effects of the inhibitors on the growth size of the two distinct spheroid-derived cell lines. (**A**) Representative images of the growth of MCF-7 before and after 24 h of treatment. (**B**,**C**) The growth rate of MCF-7 and (**D**) representative images of the growth of MDA-MB-231 in the presence of AG1478, AG1024, and their mix. (**E**,**F**) The growth rate of MDA-MB-231. Each bar represents the mean ± SD values. An asterisk (*) indicates statistically significant differences (*p* < 0.05), two asterisks indicate statistically significant differences (*p* < 0.01), and three asterisks indicate statistically significant differences (*p* < 0.001) compared to the control samples. A hash (#) indicates statistically significant differences (*p* < 0.05), two hashes indicate statistically significant differences (*p* < 0.01), and three hashes indicate statistically significant differences (*p* < 0.001) among the different samples.

The purpose of this experimental design was to investigate the effect of receptor inhibition on spheroid size. This approach aimed to simulate a more physiologically relevant tumor environment and evaluate how the inhibition of specific signaling pathways affected the tumor growth dynamics and spheroid growth potential in the untreated cells (control). To achieve this, the MCF-7 and MDA-MB-231 cells were cultured for 72 h in ultra-low-adhesion plates to form spheroids. Afterwards, the spheroids were starved and treated with the inhibitors AG1478 and AG1024 and their combination. Notably, in the case of the MCF-7 spheroids (Figure 2A), a significant and consistent reduction in the size growth rate was observed across all treatment groups, indicating strong dependence on the targeted pathways for maintaining spheroid structure and proliferative capacity. Particularly, simultaneous inhibition of both the EGFR and IGFR pathways led to the most pronounced decrease in spheroid growth (Figure 2B,C). We noticed that the MDA-MB-231 spheroids (Figure 2D) followed a similar pattern of size reduction to that for the MCF-7 spheroids; however, the magnitude of this reduction was less pronounced compared to that for MCF-7 (Figure 2E,F), with EGFR exhibiting a particularly potent effect as compared to that of IGF-IR.

### 3.3. The Dissemination of MDA-MB-231 and MCF-7 Spheroids and Their Response to TKIs

**Figure 3:**
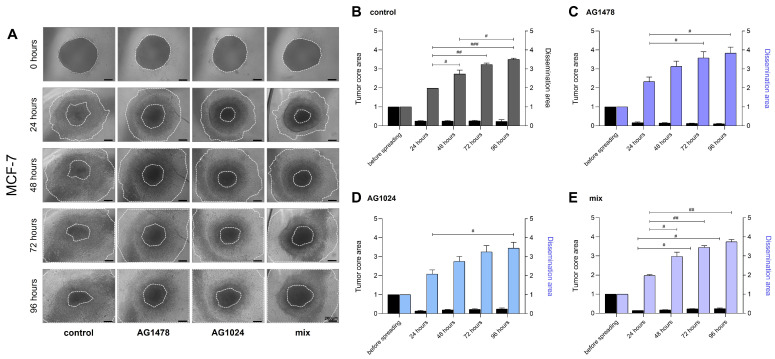
Evaluation of the effects of EGFR and IGF-IR inhibition on MCF-7 spheroid dissemination. Cells were cultured for 72 h and then were treated with AG1478, AG1024, and their mix. (**A**) Representative images of MCF-7 spheroid dissemination at time points of 0, 24, 48, and 72 h (scale bar: 200 μm; 10x magnification). Quantification graphs of the samples from the dissemination assay and the tumor core area after treatment; (**B**) the control (untreated cells), (**C**) AG1478, (**D**) AG1024, and (**E**) the mix of TKIs. Tumor core area and dissemination area were measured in inches^2^. Each bar represents the mean ± SD values from three spheroid dissemination assay analyses per condition. An asterisk (*) indicates statistically significant differences (*p*  <  0.05), and two asterisks (**) indicate statistically significant differences (*p*  <  0.01), while three asterisks (***) indicate statistically significant differences (*p*  <  0.001) compared to the control groups. A hash (#) indicates statistically significant differences (*p*  <  0.05), and two hashes (##) indicate statistically significant differences (*p*  < 0.01), while three hashes (###) indicate statistically significant differences (*p*  <  0.001) between the treatments.

One particularly interesting use of 3D spheroid models is to investigate how cells spread from spheroids while shifting from a 3D environment to a 2D substrate, mimicking the initial steps of tumor cell spreading. This dynamic process could help our understanding of how cells respond to changing environmental signals, providing vital insights into the cell morphology and motility changes across several cell lines [30]. The results revealed interesting differences between the two cell lines. First, the spheroids were transferred onto a 2D polystyrene plate and treated with the EGFR and IGF-IR inhibitors and a combination of both to assess their ability to spread for periods of 24 to 96 h (Figure 3A). The results showed that in MCF-7, the dissemination area demonstrated a distinct increase in all samples (Figure 3B–E). It is notable that under treatment with both inhibitors, there is slight expansion over time. 

**Figure 4:**
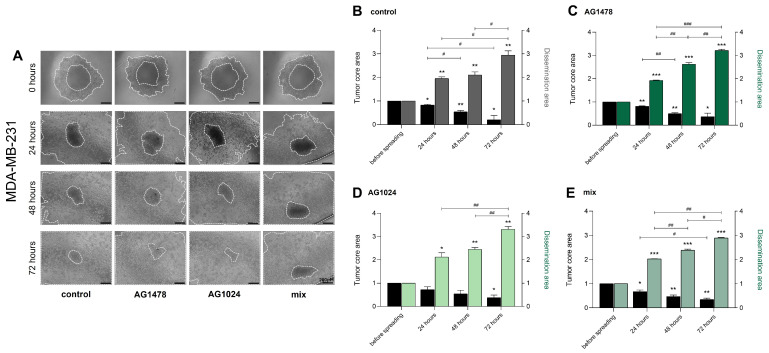
The effects of EGFR and IGF-IR inhibition on MDA-MB-231 spheroid dissemination. Cells were cultured for 72 h and then were treated with AG1478, AG1024, and their mix. (**A**) Representative images of MDA-MB-231 spheroid dissemination at the time points of 0, 24, 48, and 72 h (scale bar: 200 μΜ; magnification 10×). Quantification graphs of the samples from the dissemination assay and tumor core area after treatment; (**B**) the control (untreated cells), (**C**) AG1478, (**D**) AG1024, and (**E**) the mix of TKIs. Tumor core and dissemination area were measured in inches^2^. Each bar represents the mean ± standard deviation (SD) derived from three independent spheroid dissemination assays conducted per experimental condition. Statistical significance is denoted as follows: an asterisk (*) indicates *p* <  0.05, two asterisks (**) indicate *p*  <  0.01, and three asterisks (***) indicate *p*  <  0.001 in comparison to the corresponding control group. Additionally, statistically significant differences between treatment groups are indicated by a hash symbol (#) for *p*  <  0.05, two hashes (##) for *p*  <  0.01, and three hashes (###) for *p* < 0.001.

In contrast, in the MDA-MB-231 spheroids, the dissemination area increased progressively over time, while the tumor core decreased across all treatment conditions (Figure 4B–E). A notable morphological shift was also observed in the MDA-MB-231 cell line, as shown in Figure 4A. Specifically, these cells transitioned from an initially more epithelial-like morphology to a more typical mesenchymal phenotype. This transition highlights the highly invasive and aggressive nature of the MDA-MB-231 cells during spreading. 

### 3.4. The Migratory Capability of MCF-7 and MDA-MB-231 in 3D Conditions

**Figure 5:**
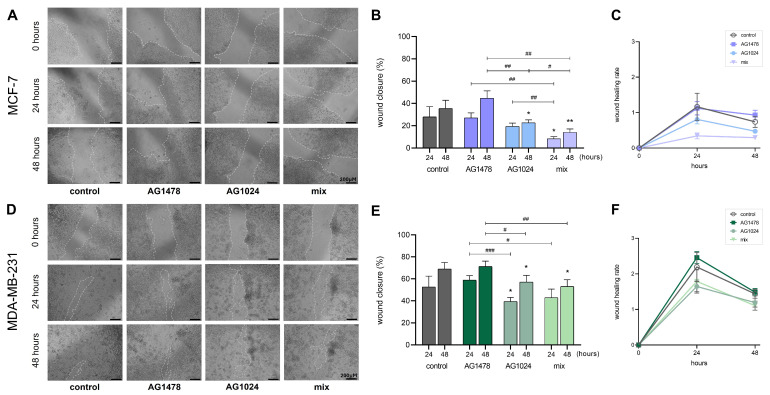
Evaluation of the effect of EGFR and IGF-IR inhibition on breast-cancer-cell-derived spheroids’ migratory capacity. The MCF-7 and MDA-MB-231 spheroids were cultured for 72 h and were then treated with the EGFR and IGF-IR inhibitors AG1478 and AG1024, respectively, as well as with their mix (AG1478 + AG1024). After, the spheroids were transferred into 2D polystyrene plates, where they were allowed to spread until they covered the well surface, and the migration of the spheroid-derived cells was examined. (**A**) Representative images of the wound healing assay of MCF-7 spheroid-derived cells at time points of 0, 24 and 48 h (scale bar: 200 μm). (**B**) A quantification graph of MCF-7 cell migration after 24 and 48 h of treatment. (**C**) Wound healing rate of MCF-7 spheroid-derived cells after 24 and 48 h of treatment. (**D**) Representative images of the wound healing assay of MDA-MB-231 spheroid-derived cells at time points of 0, 24 and 48 h (scale bar: 200 μm). (**E**) Quantification graph of MDA-MB-231 cell migration after 24 and 48 h of treatment. (**F**) Wound healing rate of MDA-MB-231 spheroid-derived cells after 24 and 48 h of treatment. In comparison to the control groups, statistically significant differences (*p* < 0.05) are indicated by an asterisk (*), and statistically significant differences (*p* < 0.01) are indicated by two asterisks (**). Statistically significant differences between the treatments are indicated by (#) if the difference is *p* < 0.05, two hashes (##) if *p* < 0.01, and three hashes (###) when *p* < 0.01.

Tumor cell migration is an essential aspect of cancer cell invasion and metastatic potential. Gaining better knowledge of these regulatory processes is critical for developing successful cancer-limiting medicines [31,32]. Taking this into account, evaluating the effects of RTK inhibitors on the metastatic potential of these cell-line-derived spheroids is pivotal. The data obtained show that the effect of inhibition followed the same pattern in both cell lines under 3D conditions. Specifically, in MCF-7 cells (Figure 5A), the inhibition of IGF-IR leads to a reduction in the wound healing rate (Figure 5B,C), and this effect is amplified by the combination of both AG1478 and AG1024. Furthermore, EGFR inhibition alone slightly affects the migration of MCF-7, although it notably contributes to a reduction in the wound healing rate in the sample with the inhibitor mix. On the contrary, the MDA-MB-231 cells demonstrate a higher wound closure rate across all treatments (Figure 5D), indicating their more motile and metastatic nature compared to that of MCF-7 (Figure 5E,F). Nonetheless, IGF-IR appears to significantly influence MDA-MB-231, evident not only from its impact on the sample involving IGF-IR inhibition but also on the sample treated with the combination of inhibitors. Subsequently, it is meaningful to examine further how this inhibition changes in 3D conditions. 

### 3.5. The MMP Gene Expression in the MDA-MB-231 and MCF-7 Spheroids

**Figure 6:**
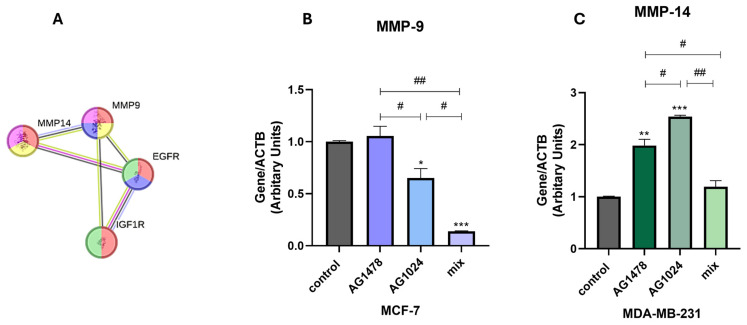
The protein–protein interaction network of EGFR and IGF-IR with MMPs and the effect of AG1478, AG1024, and their combination on the gene expression of MMPs. (**A**) The interaction network from the STRING database involving EGFR, IGF-IR, MMP-9, and MMP-14 was constructed using the ‘Multiple Proteins’ function outcome. Each node represents an individual protein (red: positive regulation of migration; blue: regulation of the EGFR and IGF-IR signaling pathways; yellow: ECM remodeling; purple: MMP activation; green: breast cancer conditions), and different line colors indicate different types of interactions (blue line: protein homology; violet line: experimentally determined interactions; light green line: text mining; dark blue line: co-expression). Real-time qPCR analysis of the mRNA levels of (**B**) MMP-9 in the MCF-7 spheroids. Real-time qPCR analysis of the mRNA levels of (**C**) MMP-14 in the MDA-MB-231 spheroids. An asterisk (*) indicates statistically significant differences (*p* < 0.05), while two asterisks (**) indicate statistically significant differences (*p*  <  0.01), and three asterisks (***) indicate statistically significant differences (*p* < 0.001) compared to the control groups. Statistically significant differences (*p* < 0.05) are indicated by a hash (#), statistically significant differences (*p* < 0.01) are indicated by two hashes (##), and statistically significant differences (*p* <0.001) between the treatments are indicated by three hashes (###).

MMPs have emerged as leading contenders due to their capacity to degrade diverse ECM components and influence cell–matrix interactions [33,34]. A bioinformatics tool like the STRING database is a useful tool, as it reveals potential biological targets and contributes to our understanding of the functional context of specific proteins, such as MMPs, in complex biological systems. An interaction network involving EGFR, IGF-IR, MMP-9, and MMP-14 was constructed using the ‘Multiple Proteins’ function in the STRING database and is depicted in Figure 6A. This representation of the protein–protein interaction network shows the way that proteins interact with the other molecules in the network but also their role in functional cellular processes. More specifically, red and blue symbolize the positive regulation of migration and the regulation of the EGFR and IGF-IR signaling pathways; yellow represents ECM remodeling; purple stands for MMP activation; and green symbolizes breast cancer conditions. Regarding the types of interactions, the blue-colored line represents protein homology, the violet-colored line indicates the experimentally determined interactions, the light-green-colored line corresponds to text mining, and the dark-blue-colored line represents co-expression. This network provides further confirmation that evaluating the role of these molecules is worthwhile.

Building on the interaction data from STRING, the expression levels of MMP-9 and MMP-14 were analyzed in a physiologically relevant context, namely 3D cultures, to assess the functional relevance of these molecules further. From Figure 6, it is evident that in MCF-7 (Figure 6B), the statistically significant reduction following the combination of the inhibitors shows the same tendency as migration, indicating the less metastatic nature of this breast cancer cell line. On the contrary, MMP-14 is significantly expressed in MDA-MB-231, independent of the treatment (Figure 6C). This upregulation may be related to the hypoxic conditions, which, in general, are known to upregulate the activity of collagenases while cells try to adapt to the low oxygen levels of the TME [35].

## 4. Discussion

In prior research conducted by our laboratory, it has been demonstrated that EGFR plays a crucial role in the proliferation of MDA-MB-231 cells [23]. Additionally, according to the transcripts per million (TPM) reported by The Human Protein Atlas [25], 61.6 TPM detected for EGFR in MDA-MB-231 cells. This observation aligns with our experiments, where we identified a significant reduction in the proliferative ability of MDA-MB-231 following EGFR inhibition, as shown with AG1478. The significance of these pathways becomes even clearer in the samples treated with a combination of inhibitors, where we observed the most substantial decrease in cell growth. In the case of the MCF-7 cells, it is noteworthy that all treatments resulted in a considerable decrease, highlighting the role of inhibition. Thus, both these results and the existing literature support the idea that EGFR is a crucial survival pathway in MDA-MB-231 cells, emphasizing its potential as a therapeutic target in this challenging TNBC model. Furthermore, the data indicates that IGF-IR signaling significantly contributes to the proliferative potential of these cells, suggesting complex involvement in tumor progression. Conversely, in the MCF-7 cells, the marked decrease observed in all experimental settings may stem from functional crosstalk between the EGFR and IGFR pathways, implying that their interplay could be vital for governing the cellular responses in ERα-positive breast cancer.

The rising need for representative models that bridged the gap between *in vitro* and clinical trials led to the development of spheroids. To more accurately replicate the in vivo tumor microenvironment, 3D cultures like spheroids provide crucial insights into the behavior of cancer cells [36,37]. These two cell lines exemplify different molecular subtypes, with MCF-7 being luminal-like and less aggressive, while MDA-MB-231 is triple-negative and exhibits high invasiveness. It is therefore reasonable to evaluate the effects of pathway inhibitors, such as those targeting EGFR and IGF-IR, which have been shown under 2D conditions to influence the proliferation of cancer cells with distinct molecular profiles, using models that more closely simulate the biological complexity of the tumor microenvironment [27]. By comparing spheroid formation and growth patterns, we examined how the inherent differences between these cell types affected the architecture and behavior of tumors in a 3D context. In the case of the MCF-7 spheroids, the consistent decrease in their growth rate, which was observed across all treatment groups, underscored significant dependence on the targeted pathways for maintaining spheroid growth potential. Specifically, the co-inhibition of both the EGFR and IGF-IR pathways led to the most marked decrease in the spheroid growth rate, suggesting that both signaling pathways were affected. A comparable trend in the size reduction was noted in the MDA-MB-231 spheroids; however, the extent of this reduction was not as significant as that in MCF-7, potentially reflecting inherent differences in pathway reliance between the two cell lines. The existing literature supports the idea that these variations can be linked to the distinct molecular and phenotypic characteristics of hormone-receptor-positive versus TNBC cells, as well as differences in their dependence on extracellular signals for survival and proliferation [38].

Observing the alterations that occur in the transition of the cells from 3D to 2D conditions offers a unique insight into cellular plasticity, with significant implications for understanding essential biological processes including metastasis, wound healing, and tissue regeneration [38]. Researchers can imitate *in vivo* conditions better by introducing 3D models into experimental processes, bridging the gap between traditional *in vitro* experiments and physiologically relevant environments. The results from the dissemination assays indicated that the area of dissemination increased in both cell lines for all treatments, while the tumor core in MDA-MB-231 decreased and it remained constant in MCF-7. The choice to focus on the 72 h time point for both cell lines was made to enable a more direct and meaningful comparison under 3D conditions. In the MDA-MB-231 spheroids, the structural integrity of the 3D model was compromised beyond 72 h due to excessive cell spreading and the morphological transition, which led to loss of the spheroid architecture. This decision was based on morphological assessments and aimed to ensure the validity of the comparisons across time points. Moreover, among the array of inhibitors used, the slight expansion over time was particularly noteworthy. These findings suggest that MCF-7 cells preserve the typical epithelial morphology, even as they spread on the culture plate. This observation points to relatively stable phenotypic behavior, in line with their less aggressive behavior and epithelial characteristics. Conversely, a marked morphological change was noted in the MDA-MB-231 cell line. These cells shifted from an initially epithelial-like appearance in the surface of 3D spheroids to the typical mesenchymal-like phenotype. This transition emphasizes the highly invasive and aggressive nature of MDA-MB-231 cells and highlights their ability to adapt phenotypically, which is a key feature of their metastatic potential.

Expanding the previous research indicating the significance of the EGFR and IGF-IR pathways in MDA-MB-231, as well as the diminished motility following treatment with either an EGFR or IGF-IR inhibitor in MCF-7 2D cell cultures [23], we employed 3D models to enhance our comprehension of the migratory potential of these two breast cancer cell lines in a setting that simulated that of in vivo tumors. The acquired results indicate that overall, a consistent pattern is exhibited in the inhibitory effect across both cell lines under 3D conditions. The reduction in the wound healing rate due to IGF-IR and the combination of treatments was more pronounced in MCF-7 than that in MDA-MB-231. Additionally, it has been established that EGFR downregulates the migratory ability of MDA-MB-231 in 2D models [22,23]. Therefore, it would be meaningful to examine how this inhibition changes in 3D conditions using additional experiments. In other words, due to these similar patterns of inhibition, further investigation into the role of EGFR is necessary, as it does not exhibit a strong inhibitory effect when used separately; thus, we cannot be certain about its contribution to the effect of the mix.

**Figure 7:**
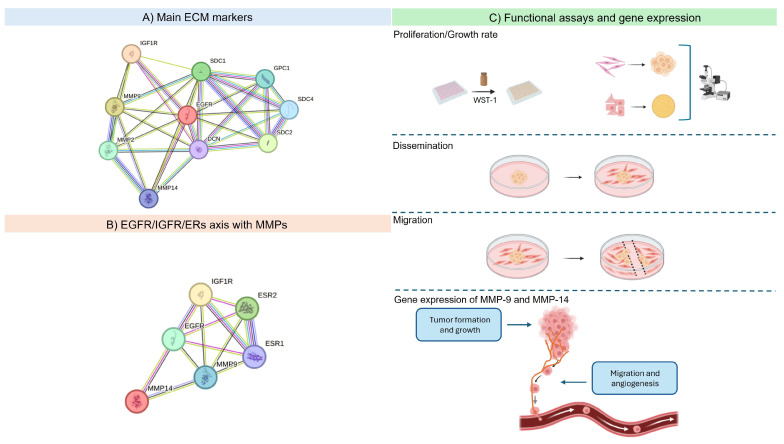
The main interaction networks among EGFR, IGF-IR, ECM components, and other receptors, along with the experimental workflow assessing their impact in breast cancer. (**A**) The known and predicted protein–protein interaction network of key ECM markers (blue line: protein homology; violet line: experimentally determined interactions; light green line: text mining; dark blue line: co-expression, light blue: from curated databases) generated utilizing the STRING database. (**B**) Physical and functional interactions (blue line: protein homology; violet line: experimentally determined interactions; light green line: text mining; dark blue line: co-expression, light blue: from curated databases) of EGFR and IGF-IR with ERs and selected MMPs. (**C**) The experimental workflow outlining the functional assays performed, as well as the key roles of MMP-9 and MMP-14, which were evaluated based on their gene expression following treatment with the TKIs in 2D and 3D conditions.

The ECM is regulated by a complex network of intricate processes and regulatory systems that function in both healthy and pathological settings. In pathological conditions, particularly in breast cancer, these mechanisms become far more complex and crucial. Structural and biochemical modification of the ECM is critical to tumor growth, invasion, and metastasis. As a result, scientists have become more interested in discovering and characterizing important molecular regulators of modified expression in the ECM, including MMPs. Based on the interaction network generated through the STRING database (Figure 6A), the functional relevance of MMP-9 and MMP-14 in cancer cell behavior was clearly demonstrated. Specifically, both MMPs were shown to influence cellular migration and modified ECM expression, findings that are well supported by the existing literature [39,40]. Under 3D culture conditions, which were assessed via gene expression experiments, the role of the MMPs was substantiated further. In particular, the downregulation of MMP-9 following dual receptor inhibition aligns with the migration and dissemination results observed in the MCF-7 cells, indicating the less metastatic nature of this breast cancer cell line. Regarding MMP-14, its expression remained notably high in the MDA-MB-231 cells, regardless of treatment. This may be attributed to the general upregulation of MMPs under hypoxic conditions, as has already been mentioned, while an alternative hypothesis suggests that it may be attributed to an alternative cellular response to the inhibitors within the 3D spheroid model. Although our inference of a hypoxic core is grounded in the well-established characteristics of spheroid models and their known impact on molecules such as MMP-14, we acknowledge that direct experimental validation would enhance the robustness of this observation. Nonetheless, there is a need for more experiments to be conducted to understand these complicated effects. The overall concept of this study along with the key experimental and functional findings, are summarized in Figure 7.

## 5. Conclusions

Breast cancer remains the most frequently diagnosed cancer in women globally, which drives ongoing research into its complex biology to improve the treatment strategies. The traditional 2D *in vitro* models used for drug screening often fall short in replicating the true nature of the TME. In contrast, 3D culture systems have emerged as more accurate and cost-effective tools that mimic tumor behavior and architecture better. This study was designed to evaluate two primary research objectives: first, to emphasize the advantages of 3D models in replicating the structure and function of breast tumors under pathological conditions, and second, to investigate the roles of RTKs—specifically EGFR and IGF-IR—in cellular behavior and modified ECM expression across two breast cancer subtypes: TNBC (MDA-MB-231) and luminal A (MCF-7). Utilizing the inhibitors AG1478 and AG1024, this study explored how these signaling pathways affect cell proliferation and ECM-related gene expression. The findings confirmed the established link between EGFR and MDA-MB-231 proliferation, as well as IGF-IR’s importance in MCF-7. Interestingly, the results also revealed an unexpected role of IGF-IR in MDA-MB-231 and evidence of EGFR/IGF-IR crosstalk in the MCF-7 cells. In the 3D MDA-MB-231 spheroids, EGFR was essential to growth, while the receptor interaction remained key to MCF-7 spheroid development. Though the dissemination assays showed no major treatment effects within each cell line, MDA-MB-231 displayed morphological changes, likely due to the environmental transition. Migration assays supported the known aggressive and mobile nature of MDA-MB-231 compared to that of MCF-7 in both 2D and 3D models. However, the specific role of EGFR in MDA-MB-231 spheroid migration requires further evaluation. Regarding the modified ECM expression, the MMP gene expression varied under 3D conditions, suggesting regulation by hypoxia, microenvironmental factors, and EMT processes. These findings underscore the importance of 3D systems for studying complex tumor behavior.

Evaluating the protein expression of ECM-based biomarkers would be valuable in future studies. Including additional cell types from the TME and utilizing matrix-based bioscaffolds could further enhance the model’s accuracy. Investigating tumor invasion under these conditions would complement the current findings on migration and dissemination. Ultimately, this work contributes to a better understanding of breast cancer progression. It also highlights the potential of targeting the EGFR and IGF-IR pathways for more effective, personalized therapies.

## Figures and Tables

**Table 1:** Primer sequences used for real-time polymerase chain reaction analysis.

Target Gene	Primer Sequences (5′-3′)		Annealing Τ (°C)
*MMP-9*	F	*TTCCAGTACCGAGAGAAAGCCTAT*	62
	R	*GGTCACGTAGCCCACTTGGT*	
*MMP-14*	F	*CATGGGCAGCGATGAAGTCT*	60
	R	*CCAGTATTTGTTCCCCTTGTAGAAGTA*	
*ACTB*	F	*TCAAGATCATTGCTCCTCCTGAG*	60
	R	*ACATCTGCTGGAAGGTGGACA*	

## Data Availability

The raw data supporting the conclusions of this article can be made available by the corresponding authors on request.

## Abbreviations

The following abbreviations are used in this manuscript:

2D/3D	Two/three-dimensional
BC	Breast cancer
ECM	Extracellular matrix
EGFR	Epidermal growth factor receptor
ERα/β	Estrogen receptor alpha/beta
HER2	Human epidermal receptor 2
IGF-IR	Insulin-like growth factor I receptor
MMPs	Matrix metalloproteinases
PR	Progesterone receptor
RTKs	Receptor tyrosine kinases
TKIs	Tyrosine kinase inhibitors
TME	Tumor microenvironment
TNBC	Triple-negative breast cancer
